# Incidence of Rabies in Humans and Domestic Animals and People's Awareness in North Gondar Zone, Ethiopia

**DOI:** 10.1371/journal.pntd.0002216

**Published:** 2013-05-09

**Authors:** Wudu Temesgen Jemberu, Wassie Molla, Gizat Almaw, Sefinew Alemu

**Affiliations:** 1 Faculty of Veterinary Medicine, University of Gondar, Gondar, Ethiopia; 2 Department of Microbiology, National Animal Health Diagnosis and Investigation Center, Sebeta, Ethiopia; The Global Alliance for Rabies Control, United States of America

## Abstract

**Background:**

Rabies is a zoonotic disease that has been prevalent in humans and animals for centuries in Ethiopia and it is often dealt with using traditional practices. There is lack of accurate quantitative information on rabies both in humans and animals in Ethiopia and little is known about the awareness of the people about the disease. In this study, we estimated the incidence of rabies in humans and domestic animals, and assessed the people's awareness about the disease in North Gondar zone, Ethiopia.

**Methodology/Principal Findings:**

The incidence of rabies in humans and domestic animals was prospectively followed up for one year period based on clinical observation. A questionnaire was also administered to 120 randomly selected dog owners and 5 traditional healers to assess the knowledge and practices about the disease. We found an annual estimated rabies incidence of 2.33 cases per 100,000 in humans, 412.83 cases per 100,000 in dogs, 19.89 cases per 100,000 in cattle, 67.68 cases per 100,000 in equines, and 14.45 cases per 100,000 in goats. Dog bite was the source of infection for all fatal rabies cases. Ninety eight percent of the questionnaire respondents were familiar with rabies and mentioned dog bite as a means of transmission. But discordant with current scientific knowledge, 84% and 32% of the respondents respectively mentioned any type of contact (irrespective of skin condition) with saliva, and inhalation as a means of transmission of rabies. Eighty four percent of the respondents relied on traditional healers for management of rabies.

**Conclusions:**

The study shows high canine rabies burden, and lack of sufficient awareness about the disease and high reliance on traditional treatment that interfere with timely post exposure management. Vaccination of dogs, proper post exposure management, and increasing the awareness of the community are suggested to reduce the disease burden.

## Author Summary

Rabies is a fatal viral disease that affects all mammals including humans. Domestic dog is the main source of rabies for humans and livestock in developing countries. Rabies has been prevalent in Ethiopia for centuries, affecting humans and livestock. In this study we estimated the incidence of the disease in North Gondar zone, Ethiopia and assessed the people's awareness about the disease. We found a high annual rabies incidence of 2.33 cases per 100,000 in humans, 412.83 cases per 100,000 in dogs, 19.89 cases per 100,000 in cattle, 67.68 cases per 100,000 in equines, and 14.45 cases per 100,000 in goats. Although almost all interviewed people were familiar with the disease, quite a lot of them (84%) have some opinions that are incongruent with existing scientific knowledge about the cause and means of transmission of the disease. We also found high reliance on traditional healers, whose practice has not been proven effective scientifically, for the management of the disease. In conclusion, the disease poses significant public health and economic problem that warrants multi-dimensional approach towards its control. Vaccination of dogs, proper post exposure management, and increasing the awareness of the community about the disease should be considered for controlling the disease.

## Introduction

Rabies is an acute encephalitis illness caused by rabies virus. Rabies virus is the prototype species of the genus *Lyssavirus* in the family of *Rhabdoviridae*. The virus affects virtually all mammals and infected species invariably die from the disease once clinical signs are manifested [1].

Rabies is endemic in developing countries of Africa and Asia, and most human deaths from the disease occur in these endemic countries [2]. Human mortality from endemic canine rabies was estimated to be 55, 000 deaths per year and was responsible for 1.74 million disability adjusted life years(DALYs) losses each year [3]. The annual cost of rabies in Africa and Asia was estimated at US$ 583.5 million most of which is due to cost of post exposure prophylaxis (PEP) [3]. Ethiopia being one of the developing countries is highly endemic for rabies. Approximately 10, 000 people were estimated to die of rabies annually in Ethiopia which makes it to be one of the worst affected countries in the world [4]. Dogs are the principal source of infection for humans and livestock [5]. In Ethiopia many households own dogs usually for guarding property. Although there are no formal studies, it is estimated that there is one owned dog per five household nationally [5]. Dog management is often poor and dog vaccination is limited to few dogs in urban centers. High population of dogs with poor management contributes for high endemicity of canine rabies in Ethiopia. In canine rabies endemic countries like Ethiopia, rabies has also significant economic importance by its effect on livestock. For example, in Africa and Asia, the annual cost of livestock losses as a result of rabies is estimated to be US$ 12.3Million [3].

In Ethiopia individuals who are exposed to rabies virus often see traditional healers for the diagnosis and treatment of the disease [5]. These widespread traditional practices of handling rabies cases are believed to interfere with timely seeking of PEP. Rabies victims specially from rural areas seek PEP treatment after exhausting the traditional medicinal intervention and usually after a loss of life from family members [5].

The available information on rabies in Ethiopia is largely based on passive reports to Ethiopian Health and Nutrition Research Institute zoonoses laboratory [6], [7], the only rabies diagnostic laboratory in the country. Passive reports usually underestimate incidence and are poor indicator of the status of the disease in countries where human and animal health information systems are inadequate [8], [9]. There is lack of accurate quantitative information on rabies both in humans and animals and little is known about the awareness of the people about the disease to apply effective control measures in Ethiopia. The objectives of the present study were, therefore, to estimate the incidence of rabies in humans and animals in North Gondar zone, Ethiopia, and assess the people's awareness of the disease in the area.

## Methods

### Ethics statement

The study was ethically reviewed and approved by Gondar University Research and Publication Office. Oral informed consent was obtained from each study participant after reading written consent form. The use of oral consent was approved by ethical committee of the office considering the fact that most of the study participants didn't read and write to give their consent in writing. The interviewers confirmed the participants' oral consent by signing on the respective consent form for each interview as per ethical review guideline of the office. The consent form mainly explains about the purpose of the study, the risks and benefits of participation in the study, conditions of confidentiality and the right to refusal or withdrawal from the study, and has a signature of the participant confirming his/her informed consent.

### Study area

The study was carried out in North Gondar administrative zone, Ethiopia. The zone's capital, Gondar town, is located at 12°35′N latitude and 37°29′E longitude. North Gondar zone has 16 districts, an area of 45,944.6 square kilometers, a human population of 2.9 million, and a livestock population of 2.4 million cattle, 2.36 million small ruminants and 0.3 million equines [10], [11].

### Sampling methods

In Ethiopia administrative zones comprise several districts and districts in turn comprise several kebeles (district sub units). Two stage cluster sampling technique was used to select sample for the study from North Gondar zone. Selection from the first cluster (districts) was done by judgment sampling and selection from the next cluster (kebeles) was done randomly. Accordingly, Gondar town district (which mainly contains town kebeles) and Dabat district (which mainly contains rural kebeles) were selected judgmentally to represent the urban and rural part of the zone, respectively. Six out of the total of 12 kebeles from Gondar town district and 10 out of the total of 29 kebeles from Dabat district were selected randomly by lottery system, and finally all households in the selected kebeles were included in the study. The human and livestock populations of the selected kebeles were taken from existing statistical sources [10], [11]. As the size of dog population was not available, census of dog population in the selected kebeles was done at the beginning of the study.

### Incidence follow up

The incidence study was done based on a prospective follow up of suspected and exposure cases of rabies for one year from April 2009 to March 2010 in the human and domestic animal populations of the selected kebeles. The rabies suspected cases were those humans and animals showing symptoms consistent with rabies (encephalitis with spasm in response to sensory stimuli, change of temper, vocalization, drooling, paralysis and other neurological signs) without a known source of exposure. Rabies exposure cases were on the other hand those animals and humans that were exposed to a known rabies suspect case. Exposure was defined according to World Health Organization (WHO) guideline [12] and included both minor exposure and severe exposure. According to the guideline, minor exposure refers to nibbling of uncovered skin, minor scratches or abrasions without bleeding while severe exposure refers to single or multiple transdermal bites or scratches, licks on broken skin, or contamination of mucous membrane with saliva of infected animals.

Follow up data were collected by resident enumerators, recruited one from each selected kebele. The enumerators were all at least high school graduates in terms of qualification. They were trained on how to do the follow up, collect the required data and the precaution to be taken when dealing with suspected cases. They conducted their data collection task under close supervision of the investigators throughout the course of the study. Before the start of the follow up, the enumerators conducted census of the dog population by going house to house in the selected kebeles and did awareness work about the study to get the cooperation of the community during the follow up. Ownerless dogs were not included in the census but their number could not be significant as the enumeration was done immediately after stray dog elimination campaign in Gondar town district, and no significant number of ownerless dogs was expected in rural kebeles of Dabat district. Once the incidence follow-up had begun the enumerators fortnightly rounded the households in the selected kebeles to note occurrence of any suspect or exposure cases and to remind the community to report any occurrence of cases in between the rounds. When cases were encountered, they were recorded with the relevant information and the follow up continued at least for 6 months after the date of their initial record. The enumerators collected data from suspect and exposures cases in a format prepared according to the WHO guideline for rabies case reporting and surveillance [13]. Human exposures were immediately referred to health organizations for appropriate measures. Final diagnosis of cases for the incidence calculation both in humans and animals was based on history of exposure, clinical signs of the disease and its fatal end. Based on WHO case classification those cases diagnosed from the follow up of suspected cases constituted suspected rabies cases and those form exposed cases constituted probable rabies cases [14]. Submission of samples for laboratory confirmation was not possible due to lack of facilities in the region.

### Questionnaire survey to assess knowledge and practices about rabies

A questionnaire that aimed at collecting information on the people's knowledge and practices about rabies was administered by face to face interview to 120 randomly selected dog owners, 10 from each kebele of Gondar town and 6 from each kebele of Dabat district. More individuals were selected from Gondar town kebeles because of their bigger population size. Additionally 5 rabies traditional healers, who were referred by the dog owner respondents as the main providers of traditional treatment against rabies in the study area, were also interviewed. The questionnaire was designed to collect information about the respondents' knowledge of the disease, its cause and means of transmission, and treatment and prevention practices. The respondents' knowledge was validated based on their description of the disease's typical clinical and epidemiological features like neurological signs, salivation, and primarily a disease of dog that is transmissible to humans and other animals.

### Data management

The data collected for both incidence and awareness study were entered into Microsoft access 2010. The data were checked for their completeness and consistency, and those incomplete and inconsistent were corrected when possible and removed otherwise. Description of the results from the cleaned data was done by descriptive statistics like percentages. The estimated incidence of the disease in humans and different species of domestic animals was calculated by dividing the clinically diagnosed cases (suspected rabies cases and probable rabies cases) by the population at risk. The incidence estimates were expressed as cases per 100, 000 individuals at risk per year

## Results

### Incidence

In the one year follow up of rabies incidence in humans and domestic animals in North Gondar zone, the highest estimated incidence was observed in dogs (412.83 cases per 100, 000 per year) followed by equines (67.68 cases per 100, 000 per year), and the estimated incidence in humans was 2.33 cases per 100, 000 per year (Table 1).

**Table 1:** Annual estimated incidence of rabies in humans and domestic animals.

Species	Suspected rabies cases	Probable rabies cases	Total No. of cases	Number at risk	Estimated incidence/100 000
**Human**	-	3	3	128146	2.33
**Dog**	12	1	13	3149	412.83
**Cattle**	2	7	9	45256	19.89
**Equines**	4	3	7	10343	67.68
**Goat**	-	2	2	13842	14.45

During the follow up period, a total of 55 cases of rabies virus exposures (32 in humans and 23 in animals) were recorded from which 16 (3 humans and 13 animals) ended with fatality. Majority (58.18%) of exposures were in humans followed by cattle (25.45%). Thirty seven (67.27%) of the exposure reports were due to bite by suspected dogs. The rest 18 (32.72%) cases were by contact of broken skin with saliva of suspected dogs and livestock, and even human in one case. Majority of exposure cases were recorded in the rural district of Dabat. Distribution of exposure cases with districts and species are presented in Table 2.

**Table 2:** Distribution of rabies virus exposure cases with district and species.

District	Species	Number exposed	Number died
**Gondar town**	Human	5	1
	Cattle	4	2
	Dog	1	1
**Dabat**	Human	27	2
	Cattle	10	5
	Horse	3	3
	Goat	3	2
	Dog	2	0
**Total**		**55**	**16**

A detail follow up of the 16 fatal rabies cases of humans and animals who were diagnosed following a known exposure history revealed that the source of exposure for all of the fatal cases was dog bite. Clinical details of the fatal human and animal cases are presented in Table 3.

**Table 3:** Clinical details of the 16 probable rabies cases of humans and domestic animals.

Species	Demography^1^	Site of bite	Severity of bite (type of exposure)	Incubation period(days)	Period of illness^2^(days)	Measures taken
**Human**	Adult female	Leg	severe	93	6	PEP, WRx,TRx
	Adult female	Head, hand	severe	35	12	PEP, TRx
	6 years girl	Belly	severe	-3	-	PEP, TRx
**Cattle**		Back	severe	38		TRx
		Back	severe	40		TRx
		Leg	minor	30		TRx
		Head	severe	15		TRx
		Leg	severe	15		TRx
		Head	minor	91		TRx
		Nose	severe	33		TRx
**Horse**		Leg	severe	23		TRx
		Leg	minor	85		TRx
		Belly	severe	36		TRx
**Goat**		Leg	severe	29		TRx
		Head	minor	25		TRx
**Dog**		Head, Ear	severe	15		No Rx

PEP: Post exposure prophylaxis using sheep brain tissue vaccine. The PEP was given within 36 hours after exposure in all three cases. The PEP was subcutaneous injection of phenolized sheep brain tissue vaccine for 14 days.

WRx: Wound treatment using soap and water.

TRx : Tradition treatment: made of herbs in humans and holy water for animals.

1 The demography for animals was ignored as it does not have much relevance with rabies virus exposure.

2 The period of illness in animals was not determined because animals showing symptoms were killed immediately by the community.

3 Missed data.

### People's knowledge and practices

Almost all (98%) of the 120 questionnaire respondents were familiar with the disease and gave it slightly different local names (e.g. ‘Kelebat’, ‘Likefit’, ‘Yebed wusha beshata’) which all mean madness. One hundred three (86%) of the respondents ascribed starvation and thirst as causes of the disease in dogs; and 8(7%) included prolonged exposure to sun heat as a cause. All of the respondents who know the disease mentioned bite as a means of transmission while 101(84%) them stated any type of contact (irrespective of the skin condition) with saliva of affected individual can transmit the disease. Thirty eight (32%) of the respondents, all of whom from Dabat district, included inhalation as means of transmission.

Regarding the practices with rabies virus exposed humans, 101 (84%) respondents used traditional medicine when they feel exposed to the disease. When it is disaggregated with district, use of traditional medicine was 100% in Dabat and 65% in Gondar town. According to the respondents, most of the traditional medicines were prepared from herbs with additional spiritual rituals in some cases, and in 20 (17%) of the cases, the treatment was preceded by diagnosis. Two of the 5 traditional healers interviewed claimed that they can diagnose whether a person is exposed or not to rabies virus, and provide such diagnostic service for their clients. The traditional healers claimed wisdom acquired from sacred scriptures as basis for their diagnosis. The respondents did not give precise information about similar treatment and diagnostic practice in animals. When it is used, it was usually holy water, and only few respondents mentioned herbs as treatment of rabies in animals.

While all respondents indicated a preventive treatment for dogs, no respondent claimed similar treatment for humans or livestock. In almost all cases the preventive treatment was constituted from herb and given for dogs by cow milk at age of 2–6 months.

Dog vaccination practice assessment revealed only 24 (20%) of the total respondents, all of them form Gondar town, vaccinate their dogs regularly. Other 13 (11%) vaccinated their dogs once in vaccination campaigns during rabies outbreaks. The rest including all respondents from Dabat district had never vaccinated their dogs against rabies. The main reasons for not vaccinating were lack of awareness about dog vaccination in Dabat district and lack access and cost of vaccine in Gondar town district.

## Discussion

### Incidences

The incidences estimated in this study were based on only clinical diagnosis of the disease. This can be considered as the limitation of the incidence study. However, because of its obvious symptoms and invariably fatal consequence, estimating the incidence of rabies based on clinical diagnosis in endemic area would not that much compromise the reliability of the estimate.

Ethiopia has been considered among the most rabies affected country in the world with an estimated annual occurrence of 10, 000 cases of human rabies which is equivalent to 18.6 cases per100, 000 people [4]. In the 1980's Bogel and Motschwillor [15] had also reported 12 cases per million people which made Ethiopia the second worse affected by rabies next to India. The present estimate of 2.33 per 100, 000 in North Gondar zone is also high and lies between the two national estimates mentioned in the preceding sentences. These previous national estimates were based on passively acquired secondary data. In developing countries accurate estimates of rabies from secondary data are difficult to obtain because of poor surveillance system and inadequate regional laboratories [16]. The incidence estimate from the present study, which is based on active follow up, would be therefore a relatively accurate estimate of the disease's burden in the study area. When compared to other incidence reports in East Africa, it is comparable to a report of 2.5 cases per 100, 000 in Kenya [8] but lower than 4.9 cases per 100, 000 reported in Tanzania [17]

Studies on the incidence of rabies in dogs in Ethiopia are nonexistent except some reports based on suspected samples that are submitted to Ethiopian Nutrition and Health Research Institute rabies laboratory [6]. In the present study a high annual incidence of about 412.83/100, 000 was determined in dogs. This estimated incidence is higher than a similar study in Chad (140/100, 000 dogs) [9] but lower than a report from Kenya (860/100, 000 dogs) [8].

Data on the incidence of rabies in livestock are limited and generally considered as a sporadic occurrence [1]. In this study, incidences of 19.89 cases per 100, 000 and 67.68 cases per 100, 000 were recorded in cattle and equines, respectively. A comparable incidence of 12.3 cases per 100, 000 in cattle was reported in agro-pastoral area of Tanzania [18]. In terms of economic impact rabies is major concern in cattle. In literatures this economic effect is considered significant in areas where bat transmitted rabies is common [1]. But in areas where the dog rabies is abundant and uncontrolled like in Ethiopia, the economic impact of rabies in livestock cannot be underemphasized. Rabies in equine could also have public health significance. During a discussion with the community in this study, the authors anecdotally learnt a story of a rabid donkey that had transmitted fatal rabies to his owner through bite.

From the total of 55 human and animal exposure cases, the 16 developed the disease and died. The rest 29 cases remained normal up to the end of their follow up duration of 6 months. This could probably be due to minor degree of exposure. It is also possible that the suspected exposing animal might not shed the virus at the time of exposure (due to intermittent shedding) or might not rabid at all. Majority of exposure cases were recorded in the Dabat rural district. This was because rural households have more livestock than urban households and some of the exposed cases in the rural district were from handling of rabies suspected livestock which increased their risk of exposure. When the details of the 16 fatal cases are seen, they were all due to dog bite confirming dog as the important reservoir and source of infection for livestock and humans. This is consistent with previous reports in Ethiopia where 95% fatal cases of human rabies were associated with dog bites [6]. In developed countries where dog rabies is controlled the main rabies cycle is associated with wild carnivores [19]. But in Ethiopia wild carnivores including the endemic Ethiopian wolf (*Canis simensis*) are threatened by spillover of virus from domestic dog [20].

The incubation periods were variable in different species and even within species which is not unexpected as the incubation period varies from few weeks to several years depending on the amount of virus in the inoculum and site of inoculation [12]. In this study incubation period of 35 days was observed in woman bitten at head region which was much shorter than 93 days for the other woman bitten on the leg. The shortest incubation period was recorded in cattle followed by in horse and then in humans (Table 3). But generally longer incubation periods were observed in livestock as compared with literature information [1], [21]. For example, the average incubation period of 37.4 days in cattle seen this study was more than double of the 15.1 days observed in an experimental study [21]. The fatal human cases were all given post exposure treatment within 36 hours using sheep brain tissue anti-rabies vaccine but it was without effect. This could be associated with many factors like vaccine quality, storage or delivery which warrants investigation.

### People's knowledge and practices

It was found that almost all respondents know the disease by slightly different names all meaning mad dog disease. Despite the fact that the community is familiar with the disease, many misconceptions about how it is caused and transmitted were observed. Although bite was correctly implicated as a means of transmission of the disease by all respondents, any direct or indirect saliva contact (irrespective of skin condition) by 84%, and inhalation by 34% of the respondents were also considered as means of transmission. Based on this conception of exposure, humans were subjected to crude traditional treatments which sometimes have serious negative consequences to their health. However a mere contact of saliva with intact skin do not pose risk of rabies virus exposure and exposures to rabies virus by inhalation are evidenced only under exceptional circumstances like inside bat caves where millions of bats congregate and create virus laden aerosol in the air [1]. Most of respondents believe that the disease in dogs is caused by starvation, thirst and prolonged exposure to sun heat. This view could be given probable explanation in relation to the notion of asymptomatic rabies carrier dogs in which stressors like starvation and thirst might induce development of clinical rabies in these carrier dogs. But the notion of asymptomatic rabies carrier dogs by itself is a contentious issue [22], [23], [24], and the association of stressors to the development of clinical rabies might be a farfetched claim.

Eighty four percent of the respondents were found to use traditional treatment following rabies virus exposure. Traditional treatment usage was more prevalent in Dabat districts than in Gondar town district indicating that people in the town use more of modern treatment because of either easy access or better awareness. While the traditional treatment is mostly made of herbs, the diagnosis is based on wisdom which the healers claimed to get it from sacred scriptures. The treatment using herbs, irrespective of its effect, is at least amenable to scientific scrutiny but the diagnosis part lacks scientific plausibility.

The use of traditional treatment by 84% of respondents shows high reliance on this unproven medication. Deressa et al. [5] noted that most fatal human rabies cases recoded in Ethiopia were mostly helped exhaustively by traditional healers. So much has to be done to reduce the high reliance of victims on traditional treatment by raising their awareness and increasing availability of post exposure anti-rabies vaccines. There are preliminary reports of anti-rabies activities of some commonly used herbs) in the traditional anti-rabies treatment practice in Ethiopia. For example, Deressa et al. [25] using mice model reported significant increase in survival time of rabies infected mice with treatment of chloroform and aqueous extracts of *Salix subserrata* leaf, and chloroform and methanol 80% extracts of *Silene macroselen* root as compared with control group. But more research is needed to identify and prove traditional medicines and practices that could be reliably used in dealing with rabies problem. Until then only scientifically proven methods of preventing the disease i.e. avoiding risk of exposure and urgent delivery of post exposure prophylaxis for those exposed should be promoted and applied.

Traditional treatment for animals was not as common as in humans. The most consistent and most surely uttered practice in animals was prophylactic treatment of puppies at young age so that they would never get the disease. This has been affirmed both by the users and the healers which again needs scientific scrutiny.

Dog vaccination practice was generally very low and totally nonexistent in rural district of Dabat. In Gondar town district, where there was better awareness, lack of access and cost of vaccine was raised as problem. Raising awareness about dog vaccination and improving access and affordability of the vaccine should be considered in control of the disease as dogs are the main reservoir of the disease.

In conclusion the study shows high incidence of rabies in both humans and animals that could be of significant public health and economic burden. Dogs are the main source of infection for both humans and livestock rabies. The lack sufficient awareness about the disease and high reliance on traditional treatment seen in this study could interfere with timely post exposure management and be an obstacle for control of the disease in the study area. In light of these findings vaccination of dogs, proper post exposure management and increasing the awareness of the community about the disease are suggested to reduce the disease burden.

## Supporting Information

Checklist S1 STROBE Checklist.
(DOCX)
