# Supplementary material for: Incidence of Rabies in Humans and Domestic Animals and People's Awareness in North Gondar Zone, Ethiopia
Source: PLoS Negl Trop Dis. 2013 May 9;7(5):e2216. doi: 10.1371/journal.pntd.0002216 (PMC3649954; doi:10.1371/journal.pntd.0002216)
Supplement: Checklist S1 — STROBE Checklist. (DOCX) [file pntd.0002216.s001.docx]

STROBE checklist

|  | Item No | Recommendation |
| --- | --- | --- |
| **Title and abstract** | 1 | 1. Indicated in the title and abstract section of the manuscript |
|  |  | (*b*) provided in the Abstract section |
| Introduction | | |
| Background/rationale | 2 | Section 1: introduction |
| Objectives | 3 | Section 1: introduction( indicated at the end of the introduction) |
| Methods | | |
| Study design | 4 | Section 2. 4 and section 2.6 |
| Setting | 5 | Section 2.2 and section 2.4 |
| Participants | 6 | (*a*) *Cohort study;* section 2.3  *Cross-sectional study*’; section 2.6 |
|  |  | (*b*) *Cohort study*; N/A |
| Variables | 7 | Section 2.4 |
| Data sources/ measurement | 8* | Section 2.4 for the follow up study  Section 2.6 for the questionnaire survey |
| Bias | 9 | N/A |
| Study size | 10 | Section 2.3 |
| Quantitative variables | 11 | N/A |
| Statistical methods | 12 | N/A |
|  |  |  |

Continued on next page

| Results | | |
| --- | --- | --- |
| Participants | 13* | Section 3.1 (table 1 and table 2) |
| Descriptive data | 14* | (a) Section 3.1 (table 1 and table 2) |
|  |  | (b) section 1 (table 2) |
| Outcome data | 15* | *Cohort study*. section 3.1 |
|  |  | *Cross-sectional study.* Section 3.3 |
| Main results | 16 | (*a*) section 3.1 and section 3.3 |
|  |  | (*b*) N/A |
|  |  | (*c*) N/A |
| Other analyses | 17 | N/A |
| Discussion | | |
| Key results | 18 | Section 4.1, section 4.2 and section 4.3 |
| Limitations | 19 | Section 4.1 |
| Interpretation | 20 | Section 4.1, section 4.2 and section 4.3 |
| Generalisability | 21 | N/A |
| Other information | | |
| Funding | 22 | Source of funding is given in article submission form |
